# Gram-Positive Bacteria Cell Wall Peptidoglycan Polymers Activate Human Dendritic Cells to Produce IL-23 and IL-1β and Promote T_H_17 Cell Differentiation

**DOI:** 10.3390/microorganisms11010173

**Published:** 2023-01-10

**Authors:** Sean Turner, Brent Raisley, Kimberly Roach, Sandra Bajaña, Melissa E. Munroe, Judith A. James, K. Mark Coggeshall, Susan Kovats

**Affiliations:** 1 Arthritis and Clinical Immunology Research Program, Oklahoma Medical Research Foundation, Oklahoma City, OK 73104, USA; 2 Department of Medicine and Pathology, University of Oklahoma Health Sciences Center, Oklahoma City, OK 73104, USA; 3 Department of Microbiology & Immunology, University of Oklahoma Health Sciences Center, Oklahoma City, OK 73104, USA

**Keywords:** peptidoglycan, dendritic cell, T helper differentiation, *Bacillus anthracis*

## Abstract

Gram-positive bacterial infections are a major cause of organ failure and mortality in sepsis. Cell wall peptidoglycan (PGN) is shed during bacterial replication, and *Bacillus anthracis* PGN promotes a sepsis-like pathology in baboons. Herein, we determined the ability of polymeric *Bacillus anthracis* PGN free from TLR ligands to shape human dendritic cell (DC) responses that are important for the initiation of T cell immunity. Monocyte-derived DCs from healthy donors were incubated with PGN polymers isolated from *Bacillus anthracis* and *Staphylococcus aureus*. PGN activated the human DCs, as judged by the increased expression of surface HLA-DR, CD83, the T cell costimulatory molecules CD40 and CD86, and the chemokine receptor CCR7. PGN elicited the DC production of IL-23, IL-6, and IL-1β but not IL-12p70. The PGN-stimulated DCs induced the differentiation of naïve allogeneic CD4^+^ T cells into T helper (T_H_) cells producing IL-17 and IL-21. Notably, the DCs from a subset of donors did not produce significant levels of IL-23 and IL-1β upon PGN stimulation, suggesting that common polymorphisms in immune response genes regulate the PGN response. In sum, purified PGN is a highly stimulatory cell wall component that activates human DCs to secrete proinflammatory cytokines and promote the differentiation of T_H_17 cells that are important for neutrophil recruitment in extracellular bacterial infections.

## 1. Introduction

Gram-positive bacteria such as *Staphylococcus aureus* comprise the majority of antibiotic-resistant strains encountered in U.S. hospitals and cause most human skin and soft tissue infections, which may be invasive and life-threatening [1]. Indeed, Gram-positive bacterial infections are present in nearly half of the sepsis patients in the U.S. and are a major cause of organ failure and mortality in sepsis [2]. Although rare, respiratory infection with the Gram-positive *Bacillus anthracis* leads to extreme bacteremia and signs of sepsis [3]. Cutaneous anthrax infections are more common in rural regions where *Bacillus anthracis* is endemic in the soil [4]. Previous studies showed that highly purified cell wall peptidoglycan (PGN) polymers isolated from Gram-positive bacteria, including *S. aureus* and *B. anthracis*, are potent inducers of pro-inflammatory cytokines in human monocytes [5,6,7]. PGN is shed during bacterial replication and is present in vegetative bacteria. Therefore, circulating polymeric PGN may play a significant role as a pro-inflammatory agent that can induce innate immune pathways in the DCs and macrophages during either cutaneous or systemic infections.

PGN, which is present in the cell walls of bacteria, is a large glycan polymer composed of alternating N-acetylglucosamine (GlcNac) and N-acetylmuramic acid (MurNac) joined by short stem peptides composed of 4–5 L- and D- amino acids [8]. Indeed, PGN is present as 90% of the dry weight of bacterial cells. PGN is stimulatory for immune cells, although the pattern recognition receptor mechanisms involved are controversial [9,10,11,12]. We built on prior work to develop protocols for the purification of PGN polymers from Gram-positive bacteria [5,6,7,11,13]. We showed that the highly purified polymeric PGN isolated from *B. anthracis* is devoid of the TLR ligands present in the unprocessed bacteria, including teichoic and lipoteichoic acid, nucleic acids, palmitoylated peptides, and polysaccharides, and is not a TLR2 ligand in the murine macrophages or TLR2-reporter HEK293 cells [14]. These highly purified *B. anthracis* PGN polymers stimulate TNFα production by human monocytes after opsonization by anti-PGN antibodies and FcγR-mediated phagocytosis, as well as other mechanisms of receptor-mediated endocytosis that deliver PGN to acidic lysosomes [15,16]. In the lysosomes, PGN polymers are digested to monomeric stem peptides, such as muramyl dipeptide (MDP) [5,6]. Upon export to the cytosol, the PGN stem peptides trigger NOD1/2 proteins, leading to the activation of NF-κB and cytokine production [7,17]. Alternately, acetylated forms of PGN can be detected by hexokinase, which leads to NLRP3 inflammasome activation [18]. Human monocytes show greater activation and cytokine production after stimulation with PGN polymers compared to stimulation with muropeptides, such as MDP, or soluble PGN digestion products [7]. 

In addition to the induction of monocyte inflammatory cytokine responses, PGN may also activate the adaptive immune response mediated by the dendritic cells (DCs). In peripheral tissues or lymph nodes, DCs (resident or descended from infiltrating inflammatory monocytes) are exposed to vegetative bacteria, shed PGN, or PGN-derived muropeptides released during growth and cell wall recycling [6,19]. DCs respond to bacterial molecules by increasing the expression of MHC class II and T cell costimulatory molecules and producing soluble cytokines and chemokines. The complement of the cytokines produced by the activated DCs determines their ability to polarize particular subsets of CD4^+^ T cells (T_H_). DC production of IL-12p70 leads the T_H_1 cells to produce IFNγ, which is important for the clearance of viral infections, while DC production of IL-23 and IL-1 leads the T_H_17 cells to produce IL-17, which is important for the clearance of extracellular bacterial infections [20]. While it was reported that the combination of MDP and TLR2 ligands, as well as commercially available *S. aureus* PGN, leads to activation of human DCs such that they produce IL-23 and activate memory T_H_17 cells [21,22,23], the ability of polymeric PGN free from TLR ligands, such as PGN from *B. anthracis*, to shape human DC responses that are important for the initiation of T cell immunity remain unknown.

Herein, we tested the hypothesis that highly purified PGN polymers isolated from *B. anthracis* and lacking TLR2 agonist activity would activate human-monocyte-derived DCs. We report that PGN polymers activate DCs, leading to the upregulation of the T cell costimulatory molecules CD40 and CD86, as well as CCR7, HLA-DR and CD83. PGN-activated DCs produce IL-23, IL-1β and IL-6 but not IL-12p70. This cytokine profile enables the DCs to stimulate the differentiation of naïve CD4^+^ T cells into T_H_ cells producing IL-17. Thus, PGN-exposed DCs may promote the differentiation of T_H_17 cells that are important for neutrophil recruitment in extracellular bacterial infections. In addition, the secretion of IL-6 and IL-1β by PGN-stimulated DCs may contribute to sepsis pathology when bacteria and PGN reach high levels in the blood or tissues.

## 2. Materials and Methods

### 2.1. Preparation of Bacillus anthracis and Staphylococcus aureus peptidoglycan (PGN) and Heat-Killed Bacteria (HKB)

PGN was isolated, as described, from *B. anthracis* (Δ Sterne strain) and *S. aureus* (strain MN8) [14]. The *B. anthracis* Δ Sterne strain, NR-9401, was obtained from the NIH NIAID BEI Resources. The *S. aureus* strain MN8, NR-45918, was provided by the Network on Antimicrobial Resistance in *Staphylococcus aureus* (NARSA) for distribution by BEI Resources, NIAID, NIH. Briefly, the *B. anthracis* and *S. aureus* vegetative bacteria were collected after overnight culture and boiled in 8% SDS in water. The crude cell wall was digested with DNase and RNase, followed by digestion with proteinase K. Hydrofluoric acid was used to remove traces of teichoic acids and was unlikely to alter the sugar acetylation, as most of the PGN in *B. anthracis* is naturally de-acetylated [24]. The PGN purity and concentration were determined by amino acid analysis. As reported, the *B. anthracis* polymeric macromolecules had a median length of 0.268 μM and width of 0.205 μM [16]. The chemical composition of the muropeptides after PGN digestion was as reported: *B. anthracis* was identified as the DAP-type PGN that activates NOD1 and NOD2, and *S. aureus* was identified as the Lys-type PGN that can activate NOD2 [7,14]. Prior work showed that this purification protocol led *B. anthracis* PGN to be devoid of TLR2 ligands, while the *S. aureus* PGN contained contaminating lipopeptides with TLR2 activity when assayed on murine macrophages [14]. To generate HKB, bacteria were incubated for 1 h in a 70 °C water bath, followed by plating on solid medium to confirm that the cells were killed. Both the HKB and PGN were washed with endotoxin-free water and sonicated at 4 W for 10 min to yield a more uniform suspension prior to their addition to the cells. The amounts of HKB added to the cells, as reported in the figures, were based on calculations of the PGN equivalents in the whole bacteria. Typically, 10 μg/mL PGN corresponded to 10^7^ cfu/mL HKB [25]. 

### 2.2. Generation of Monocyte-Derived Dendritic Cells

Heparinized peripheral blood was obtained from healthy volunteers (male and female genders) with written informed consent, according to a protocol approved by the OMRF Institutional Review Board. Leukocyte buffy coats provided by anonymous donors were purchased from the Oklahoma Blood Institute. PBMC were isolated using Lymphocyte Separation Medium gradients (Mediatech Inc., Manassas, VA, USA). The CD14^+^ monocytes were isolated by negative selection using an EasySep human monocyte enrichment kit (Stem Cell Technologies, Vancouver, BC, Canada). The monocytes were cultured at 10^6^/mL in RPMI, including 10% FCS with 30 ng/mL GM-CSF and 20 ng/mL IL-4 (recombinant cytokines from Peprotech, Rocky Hill, NJ, USA), for 6 days to promote DC differentiation. The differentiated DCs were CD14^–^ CD11c^+^ CD209^+^ HLA-DR^+^.

### 2.3. Assessment of DC Activation

On day 6 after differentiation was initiated, the DCs were harvested, plated at 5 × 10^5^/mL and left unstimulated or stimulated with purified and sonicated PGN (1–100 µg/mL), HKB (1–10 µg/mL PGN equivalents) or LPS (100 ng/mL) + IFNγ (2000 IU/mL) in the presence of 5% pooled human AB serum (Innovative Research Inc). In the preliminary experiments (*not shown*), we observed reduced DC responses when the human serum was omitted, consistent with our prior work showing the monocyte FcγR-mediated uptake of PGN bound to the anti-PGN antibodies present in human serum [15]. Therefore, pooled human serum that was shown to contain antibodies that bind both sources of PGN [15] was included in the experiments discussed here. After 18 h, the DCs were assessed for changes in cell surface markers using flow cytometry. For the measurement of the secreted cytokines at 24 h after stimulation, DC supernatants were collected from the duplicate wells, each containing 50,000 DCs.

### 2.4. mAbs and Flow Cytometry

The cells were pre-incubated with human FcγR-binding inhibitor (eBioscience, San Diego, CA, USA) and 2% human serum and labeled with optimally titered mAbs in FACS buffer (PBS, 5% newborn calf serum, 0.1% sodium azide). The DCs were stained with 6–7 parameter combinations of fluorochrome-labeled mAbs specific to CD14 (clone M5E2), CD11c (BL46), CD209 (9E9A8), HLA-DR (L243), CCR7 (G043H7), CD40 (5C3), CD86 (IT2.2) and CD83 (HB15e) (obtained from eBioscience, BD Biosciences, San Jose, CA, USA, or Biolegend, San Diego, CA, USA). The T cells were stained with mAbs specific to CD4 (OKT4), CD45RA (HI100) and CD45RO (UCHL1). The samples were run on an LSRII instrument (BD Biosciences) and the data were analyzed with FlowJo (TreeStar Inc., Ashland, OR, USA) software.

### 2.5. Allogeneic T Cell Assays

Naïve CD4^+^ CD45RA^+^ CD45RO^–^ T cells were isolated using an EasySep human naïve T cell kit (Stem Cell Technologies) with a purity of 95%. The T cells (40,000 per well) were incubated with allogeneic DCs (3000 per well) in triplicate on round-bottomed 96-well plates for 7 days. The T cells remained viable and increased in number over the 7 days, as judged by the observation of the culture wells with an inverted microscope. On day 7, the T cells were stimulated for 16 h with 10 ng/mL phorbol 12,13-dibutyrate (PDBU) and 200 ng/mL ionomycin, after which the supernatants were collected for the measurement of the secreted cytokines.

### 2.6. Cytokine Assays

The cytokines secreted by the DCs (IL-12p70, IL-23p19, IL-6 and IL-1β) and T cells (IL-17, IL-21, IFNγ) were measured using xMAP multiplex assays (Affymetrix/eBioscience, Santa Clara, CA, USA) at the OMRF Serum Analyte and Biomarker core facility, as previously described [26].

### 2.7. Statistics

The statistical analyses were performed using Prism GraphPad software and are indicated in the figure legends. The data involving the surface markers on the multiple-donor DCs were analyzed using repeated measure one-way ANOVAs, followed by multiple comparison tests. The data involving the cytokine measurements of the responder individuals were log-transformed prior to Friedman’s ANOVA analyses and Dunn’s multiple comparison tests. The significance of the differences in the cytokine values obtained from responders and non-responders for each stimulus were evaluated using Mann–Whitney tests.

## 3. Results

### 3.1. Peptidoglycan Polymers Isolated from Gram-Positive B. anthracis Activate Human Dendritic Cells

We generated a preparation of highly purified PGN polymers from *B. anthracis* vegetative bacteria (ΔSterne strain). This PGN preparation lacks the TLR-stimulating molecules (teichoic and lipoteichoic acid, palmitoylated proteins, polysaccharides, nucleic acids) present in native bacteria. The preparation also lacks LPS, which may be introduced during PGN isolation procedures. Notably, the purified *B. anthracis* PGN polymers do not activate NF-κB signaling in TLR2-transfected HEK293 cells, nor do they stimulate murine TLR2^+^ macrophages, indicating the absence of a TLR2 ligand [14].

**Figure 1:**
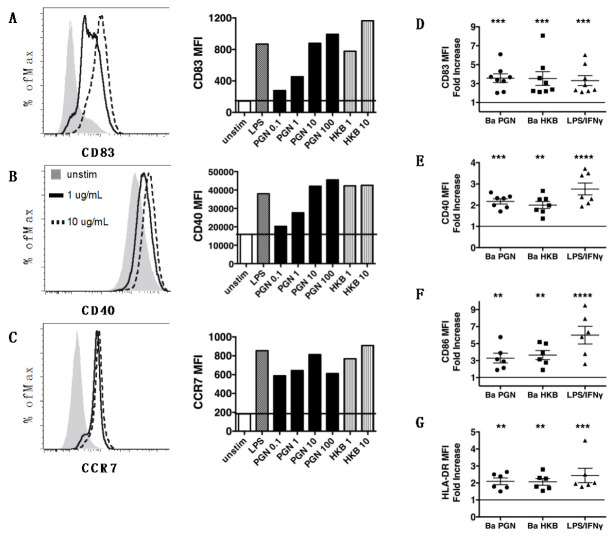
*B. anthracis* PGN and heat-killed bacteria stimulate human DCs to increase the surface expression of proteins that are important for the migration and activation of T cells. (**A**–**C**) DCs were left unstimulated (unstim) or stimulated for 18 h with LPS (100 ng/mL) + IFNγ (2000 IU/mL), *B. anthracis* PGN (0.1–100 μg/mL) or *B. anthracis* HKB (1–10 μg/mL PGN equivalents) in the presence of 5% human serum. The binding of mAbs specific to (**A**) CD83, (**B**) CD40 and (**C**) CCR7 on DCs exposed to 0, 1 or 10 μg/mL PGN is shown (legend in panel B), and the mean fluorescence intensity (MFI) of the expression for each condition tested is plotted in the corresponding bar graph. The titration data are representative of two donors in independent experiments. (**D**–**G**) Shown above is the fold increase in (**D**) CD83, (**E**) CD40, (**F**) CD86 and (**G**) HLA-DR on the DCs exposed to the stimuli indicated on the x-axis relative to the unstimulated DCs, for which the values were set to 1. The DCs were incubated with *B. anthracis* (Ba) PGN (10 µg/mL, circles) and HKB (10 µg/mL, squares) or LPS/IFNγ (triangles). For each stimulus, the symbols represent individual donors (n = 6–8), and the mean and SEM are indicated. The significance of the differences was evaluated using a repeated measure one-way ANOVA followed by a Dunnett’s multiple comparison test to compare the mean of the unstimulated control with that of each stimulated condition. The *p*-values for each stimulated DC response, relative to the unstimulated DCs, are indicated by **, *p* < 0.01; ***, *p* < 0.001; ****p < 0.0001.

The exposure of human-monocyte-derived DCs to *B. anthracis* PGN (0.1–100 μg/mL) resulted in dose-dependent DC activation (Figure 1A–C). The PGN-exposed DCs increased the surface display of CD83, an activation-induced molecule that fosters the stable surface expression of MHCII and CD86 (Figure 1A), the T cell costimulatory molecule CD40 (Figure 1B) and the chemokine receptor CCR7 that directs migration to the lymph nodes (Figure 1C). PGN at 10 μg/mL induced a maximal response, without reducing the viability, and this concentration was used in our subsequent experiments. The DC activation elicited by the PGN was comparable to that elicited by the *B. anthracis* heat-killed bacteria (HKB) present at 10 μg/mL as a PGN equivalent (Figure 1A–C). PGN was not as potent as *E. coli* LPS; the response to 10 μg/mL PGN was comparable to 0.1 μg/mL LPS (Figure 1A–C).

The DCs from all the human donors tested were capable of responding to *B. anthracis* PGN and HKB by increasing the surface expression of CD40, CD83, CD86 and HLA-DR (Figure 1E–H). This response was comparable to that triggered by LPS/IFNγ. Taken together, these data show that human DCs respond to the purified PGN from Gram-positive bacteria by increasing the surface expression of MHC class II and costimulatory molecules and chemokine receptors that are important for DC migration to the lymph nodes and interactions with the T cells.

### 3.2. B. anthracis PGN-Activated DCs Produce IL-23 and IL-1β but Not IL-12p70

**Figure 2:**
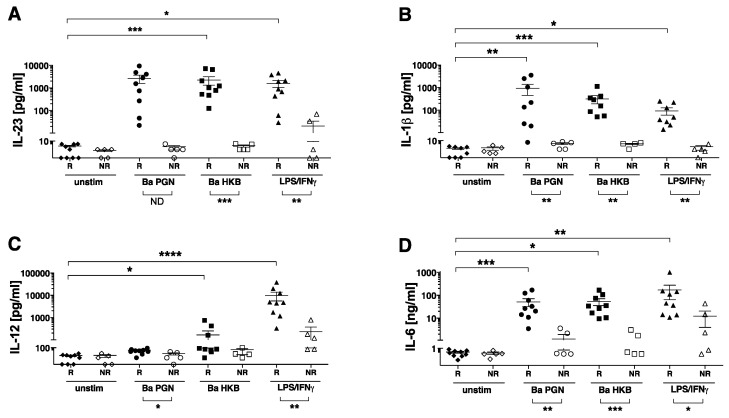
*B. anthracis* PGN stimulates human DCs to secrete IL-23, IL-6 and IL-1β but not IL-12p70. (**A**–**D**) DCs were left unstimulated (unstim) (diamonds) or stimulated for 24 h in duplicate with LPS (100 ng/mL) + IFNγ (2000 IU/mL) (triangles), *B. anthracis* (Ba) PGN (10 μg/mL) (circles) or *B. anthracis* HKB (10 µg/mL PGN equivalent) (squares) in the presence of 5% human serum. The cytokines present in cell supernatants were quantified using xMAP multiplex assays. The individual donors were divided into responders (R, closed symbols) and non-responders (NR, open symbols) based on the PGN-induced production of IL-23. The NR were defined as those whose Ba PGN-stimulated DCs produced IL-23 in amounts less than the mean + 3 SD (0.464 + 2.32 pg/mL) of their unstimulated DCs. The mean + SD of the 14 unstimulated samples (all <2 pg/mL) was 0.464 + 0.775; the mean + SD of the 5 NR samples was 0.46 + 0.862 (all <2 pg/mL); and the mean + SD of the 9 R samples was 2642 + 3098 (range 22–9405 pg/mL). Symbols represent the averaged values obtained by assaying duplicate wells for each stimulus of the DCs from individual donors (n = 14). The significance of the differences between the responders (excluding the Ba PGN-induced IL-23 measurement used to define the R and NR groups) was evaluated using a Friedman’s ANOVA of log-transformed data, followed by Dunn’s multiple comparison test to compare the mean of the unstimulated control with each stimulated condition. The significance of the differences between the responders and non-responders for each stimulus (excluding the Ba PGN-induced IL-23 measurement used to define the R and NR groups) was evaluated using a Mann–Whitney test (*p*-values are indicated below the x-axis). The *p*-values are indicated by *, *p* < 0.05; **, *p* < 0.01; ***, *p* < 0.001; **** *p* < 0.0001. ND, not determined.

To determine the cytokines produced by the PGN-stimulated DCs, we measured the levels of IL-12p70, IL-23, IL-6 and IL-1β in the DC culture supernatants collected 24 h after stimulation with *B. anthracis* PGN (Figure 2). In our analyses of the DCs generated by the 14 donors, we found a striking individual variation in the magnitude of the cytokine response to PGN. The *B. anthracis* PGN-stimulated DCs from ~60% of the donors (9/14) produced significant amounts of IL-23 (labeled as R, responders, based on the IL-23 levels, which were greater than the mean + 3 SD of the negligible IL-23 levels produced by the unstimulated DCs) (Figure 2A). In contrast, the *B. anthracis* PGN-stimulated DCs from the other donors (5/14) did not produce any detectable IL-23 compared to the unstimulated DCs (labeled as NR, non-responders) (Figure 2A). Similarly, while the R group produced significant amounts of IL-1β, the NR group did not produce IL-1β in levels above the unstimulated DCs (Figure 2B). The PGN-exposed responder DCs did not produce IL-12p70 (Figure 2C). This was in notable contrast with the DCs stimulated by LPS/IFNγ, which produced both IL-23 and IL-12p70 (Figure 2A,C). PGN also elicited the DC production of IL-6 (Figure 2D). For the individuals not producing IL-23, the amount of PGN-induced IL-6 was ~10-fold lower than that of the IL-23-producing responders, suggesting that the overall magnitude of the response to PGN was much lower. *B. anthracis* HKB also induced the production of IL-23, IL-6 and IL-1β and significantly less IL-12p70- than LPS/IFNγ-stimulated DCs (Figure 2A–D), and again, the donors fell into the same groups of IL-23-producing responders or non-responders, as defined for PGN.

Thus, PGN isolated from *B. anthracis* elicited the DC production of IL-23, IL-6 and IL-1β but not IL-12. Robust DC cytokine production in response to PGN occurred in ~60% of the donors studied, indicating significant donor variation in the ability to respond to PGN. The response to LPS/IFNγ was also decreased among the PGN non-responders. The DCs from all the donors could respond to PGN by increasing the surface expression of the costimulatory molecules CD86, CD40 and CD83 (Figure 1), and no correlation was found between the magnitude of the marker surface expression and the ability to produce IL-23.

### 3.3. B. anthracis PGN-Activated DCs Induce Naïve Allogeneic CD4^+^ T Cells to Produce IL-17

**Figure 3:**
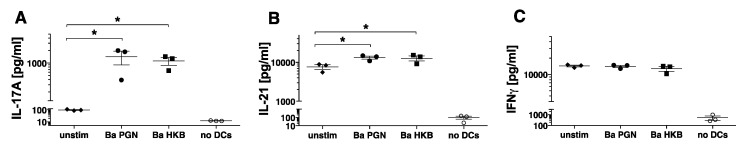
*B. anthracis* PGN-stimulated DCs induce naïve allogeneic CD4^+^ T cells to produce IL-17 and IL-21. Isolated allogeneic naïve CD4^+^ T cells were incubated with DCs that were left unstimulated (unstim) (diamonds) or stimulated for 18 h with *B. anthracis* (Ba) PGN (10 μg/mL) (circles) or *B. anthracis* HKB (10 μg/mL PGN equivalents) (squares) in the presence of 5% human serum. T + DC cultures were set up in triplicate, and the control wells contained T cells without DCs. After 7 days, the T cells were restimulated with PDBU/Ionomycin, and 16 h later, the supernatants were collected and assayed for (**A**) IL-17A, (**B**) IL-21 and (**C**) IFNγ using xMAP multiplex assays. The symbols represent the average of the triplicate wells for the individual donors (n = 3). The significance of the differences was evaluated using a one-way ANOVA, followed by a multiple comparison test. The *p*-values for each T cell response to the stimulated DCs, relative to the T cell response to the unstimulated DCs, are indicated by *, *p* < 0.05.

The complement of the cytokines produced by activated DCs determines their ability to polarize particular subsets of T_H_ cells. We therefore hypothesized that PGN-stimulated DCs secreting IL-23, IL-6 and IL-1β would induce the polarization of naïve T cells into T_H_17 cells. To test this, we incubated the stimulated DCs with allogeneic naïve CD4^+^ CD45RA^+^ T cells for 7 days. To determine whether the CD4^+^ T cells had become polarized, the T cells were stimulated with PDBU/Ionomycin, and the levels of the secreted cytokines (IFNγ, IL-17, IL-21) were measured. Here, we report on the T cell responses of three donors whose DCs produced IL-23 in response to PGN. The DCs exposed to *B. anthracis* PGN and HKB induced the naïve T cells to differentiate into T_H_ cells producing IL-17 and IL-21 (Figure 3A,B). The T cells incubated with unstimulated DCs produced IFNγ, and this was unchanged by the stimulation of the DCs (Figure 3C). Unstimulated DCs may produce other factors that induce IFNγ production by naïve T cells, as reported in [21], and this was not altered by PGN or HKB stimulation. In the assays performed on donor DCs that did not produce IL-23, we identified neither the T cell production of IL-17 nor changes in baseline IFNγ production (*data not shown*). Thus, the DCs incubated with *B. anthracis* PGN or HKB induced the naïve CD4^+^ T cells to differentiate into T_H_ cells producing IL-17 and IL-21.

### 3.4. S. aureus PGN Stimulates Human DC Production of IL-23 and IL-1β, Resulting in their Ability to Promote T_H_17 Differentiation

**Figure 4:**
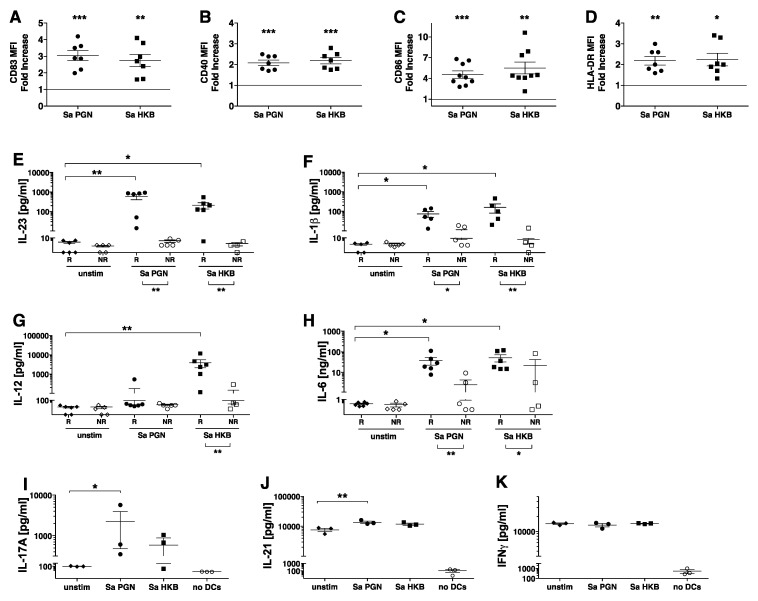
*S. aureus* PGN stimulates human DC production of IL-23 and IL1β, resulting in their ability to promote naive T_H_17 differentiation. (**A**–**D**) DCs were incubated with *S. aureus* (Sa) PGN (circles) or HKB (squares) (10 μg/mL). Shown above is the fold increase in (**A**) CD83, (**B**) CD40, (**C**) CD86 and (**D**) HLA-DR on the DCs exposed to the stimuli indicated on the x-axis, relative to the unstimulated DCs, for which the values were set to 1. For each stimulus, the symbols represent individual donors (n = 7–9), and the mean and SEM are indicated. The significance of the differences was evaluated using a repeated measure one-way ANOVA followed by Tukey’s multiple comparison test to compare the mean of the response of the unstimulated DCs with the response of the stimulated DCs. (**E**–**H**) DCs were left unstimulated (unstim) (diamonds) or stimulated for 24 h with Sa PGN (10 μg/mL) (circles) or HKB (10 μg/mL PGN equivalent) (squares) in the presence of 5% human serum. The cytokines, including (**E**) IL-23 (**F**) IL-1β (**G**) IL-12p70 and (**H**) IL-6, present in the cell supernatants were quantified using xMAP multiplex assays. The individual donors were divided into responders (R, closed symbols) and non-responders (NR, open symbols) based on the Ba PGN-induced production of IL-23, as described in Figure 2. The symbols represent the averaged values obtained by assaying duplicate wells for each stimulus of the DCs from individual donors (n = 10). The significance of the differences between the responders was evaluated using a Friedman’s ANOVA of log-transformed data followed by Dunn’s multiple comparison test to compare the mean of the unstimulated control with each stimulated condition. The significance of the differences between the responders and non-responders for each stimulus was evaluated using a Mann–Whitney test (*p*-values are indicated below the x-axis). (**I**–**K**) Isolated allogeneic naïve CD4^+^ T cells were incubated with the DCs that were left unstimulated (unstim) (diamonds) or stimulated for 18 h with Sa PGN (10 μg/mL) (closed circles) or HKB (10 μg/mL PGN equivalent) (squares) in the presence of 5% human serum. T + DC cultures were set up in triplicate, and the control wells contained T cells without DCs (open circles). After 7 days, the T cells were restimulated with PDBU/Ionomycin, and 16 h later, the supernatants were collected and assayed for (**I**) IL-17A, (**J**) IL-21 and (**K**) IFNγ using xMAP multiplex assays. The symbols represent the average of the triplicate wells for the individual donors (n = 3). The significance of the differences was evaluated using a one-way ANOVA followed by a multiple comparison test to compare the mean of the response to the unstimulated DCs with the response to the stimulated DCs. In all the panels, the *p*-values are indicated by *, *p* < 0.05; **, *p* < 0.01; ***, *p* < 0.001.

To determine whether other PGN archetypes can similarly stimulate DCs, we tested PGN macromolecules isolated from *S. aureus* (strain MN8), which harbor a lysine-containing stem peptide and contain lipopeptide anchors with TLR2 immunostimulatory activity [14,27]. *S. aureus* PGN and HKB activated DCs from multiple donors to increase surface expression of CD83, CD40, CD86 and HLA-DR (Figure 4A–D). *S. aureus* PGN induced DC production of IL-23, IL-6 and IL-1β but not IL-12p70 (Figure 4E–H). We used the definition of the responder and non-responder groups in Figure 2A for *B. anthracis* PGN. The *S. aureus* PGN-stimulated DCs from individuals in the responder group produced IL-23 in amounts greater than the unstimulated DCs. The IL-23 responders also produced IL-1β and IL-6. The non-responder individuals showed low responses to both *S. aureus* and *B. anthracis* PGN. Unlike the *B. anthracis* HKB, the *S. aureus* HKB induced a substantial production of both IL-23 and IL-12p70 (Figure 4E,G).

The DCs exposed to *S. aureus* PGN induced the naïve CD4^+^ CD45RA^+^ T cells to produce IL-17 and IL-21 (Figure 4I,J). A similar trend was observed for *S. aureus* HKB (Figure 4I,J). The T cells incubated with unstimulated DCs produced IFNγ, and this was unchanged by the stimulation of the DCs, even when they were stimulated by *S. aureus* HKB, which induces IL-12p70 production (Figure 4K).

In sum, despite the differences in the structure of the PGN stem peptides, both *S. aureus* and *B. anthracis* PGN and HKB stimulated the DCs to produce IL-23 and IL-1β, which led to their ability to induce the naive CD4^+^ T cells to differentiate into T_H_ cells producing IL-17 and IL-21.

## 4. Discussion

Humans are typically exposed to *Bacillus anthracis* spores by the cutaneous or pulmonary route. The spores germinate to vegetative bacteria, which then replicate rapidly and disseminate throughout the host, often resulting in a high density (10^8^ CFU/mL) in the blood [28]. In peripheral tissues or lymph nodes, resident DCs, as well as DCs derived from infiltrating inflammatory monocytes, are exposed to vegetative bacteria germinating from spores. The *B. anthracis* toxins, the lethal toxin and edema toxin, inhibit DC activation and immune responses [29,30]. However, the infection of animals with the ΔSterne strain of *B. anthracis* (lacking toxins and capsule) leads to sepsis [31], indicating that components of the vegetative bacteria can induce significant inflammation. Indeed, the infusion of *B. anthracis* PGN alone induces a sepsis-like pathophysiology, including disseminated intravascular coagulation and multiple organ failure in baboons [32]. PGN is a strong inducer of inflammation and is shed during bacterial replication and accumulates to reach significant levels in the blood and tissue. PGN-derived muropeptides may also be released during growth and cell wall recycling [19]. In earlier studies, we showed that *B. anthracis* PGN polymers activate human monocytes and neutrophils to produce TNFα and IL-8 [6] and induce human platelets to aggregate and express prothrombinase activity [33].

Herein, we interrogated the innate responses initiated by human DCs upon exposure to *B. anthracis* and *S. aureus* PGN polymers and the parental heat-killed bacteria. The highly purified PGN polymers activated the DCs, leading to the upregulation of the T cell costimulatory molecules CD40 and CD86, as well as CCR7, HLA-DR and CD83. The PGN-activated DCs produced IL-23, IL-1β and IL-6 but not IL-12p70. The PGN-exposed DCs producing IL-23 and IL-1β promoted the differentiation of naïve CD4^+^ T cells into T_H_ cells producing IL-17 and IL-21. In contrast, prior work showing that MDP alone is not stimulatory for human DCs, and that costimulation with a TLR2 ligand is necessary to induce IL-23 production [21,34]. This suggests that the free muropeptides generated during bacterial growth will not stimulate DCs independently. Here, we showed that *B. anthracis* PGN polymers lacking TLR2 agonist activity stimulated DCs, indicating that TLR2 priming is not necessary for PGN to induce DC responses capable of directing naive T cell differentiation into T_H_17 cells.

In addition to high levels of circulating PGN, the DC-mediated T cell response is driven by the complement of the pathogen-associated molecular patterns (PAMPs) present in the entire bacteria. Reports show that additive signaling through the MDP/NOD2 axis and either TLR4 or TLR2 increases the DC production of IL-12 and T cell priming capacity [35,36,37]. Thus, the DC production of IL-12 elicited by the heat-killed bacteria in our study is likely due other PAMPs present in the whole bacterium. Similarly, NOD activation by free muropeptides may be optimized by concurrent PAMP signaling in vivo. Interestingly, the donors with poor responses to the purified PGN also showed low responses to the heat-killed bacteria, suggesting that the DC response to heat-killed bacteria is dominated by PGN, despite the presence of other PAMP ligands for innate sensors, such as TLRs.

Our work is consistent with reports showing that human DCs exposed to germinating *B. anthracis* spores produce IL-23 and induce T_H_17 activation [38]. Similarly, our findings complement reports showing that commercially available *S. aureus* PGN promotes IL-23 production, leading to T_H_17 differentiation [22,23]. Taken together, our data and those of other authors indicate that PGN-exposed DCs promote the differentiation of T_H_17 cells that are important for neutrophil recruitment in Gram-positive bacterial infections. Interestingly, neutrophil infiltrates characterize cutaneous [39] but not inhalational anthrax [40], and Th17 responses have been reported in natural cutaneous anthrax infections in humans [41]. In addition, the DC production of pro-inflammatory IL-6 and IL-1β upon PGN stimulation could exacerbate the inflammatory sepsis pathology in instances when the bacteria and PGN reach high levels in the blood or tissues.

Monocytes and DCs generated by healthy human donors exhibit significant variability in the magnitude of cytokine production and innate immune signaling in response to the stimulation of pattern recognition receptors [42,43,44]. Common polymorphisms in genes regulating innate immune responses (e.g., *IRF5*, *NOD2*, *CARD9*) are present in populations with significant allele frequency. These common genetic variants regulate the extent of gene expression during pathogen-sensing innate immune responses [42,43,44]. For example, common *IRF5* polymorphisms contribute to the individual variability in the magnitude of cytokine production induced by the NOD2 and TLR ligands in human monocyte-derived DCs, and *IRF5* alleles associated with autoimmunity lead to increased cytokine secretion [42]. DCs generated by Crohn’s Disease patients with homozygous NOD2 mutations have a reduced capacity to produce IL-23 and induce T_H_17 when primed with TLR2 ligands in addition to MDP [21]. In our study, the individual donors with low responses to PGN isolated from *B. anthracis* or *S. aureus* may harbor one or more alleles of the innate immune genes that are associated with the reduced production of cytokines. Individual donors may also harbor polymorphic alleles that impact the pathways of PGN internalization [16]. The individual variation in cytokine synthesis, but not costimulatory molecule upregulation, documented in our study may also indicate that a higher threshold of stimulation is required for signaling pathways that are important for cytokine gene synthesis. Variation in the magnitude of the innate responses of myeloid cells to PGN likely governs the course of immunity during Gram-positive bacterial infections.

## References

[1] Miller L.S., Cho J.S. (2011). Immunity against Staphylococcus aureus cutaneous infections. Nat. Rev. Immunol..

[2] Martin G.S., Mannino D.M., Eaton S., Moss M. (2003). The epidemiology of sepsis in the United States from 1979 through 2000. N. Engl. J. Med..

[3] Coggeshall K.M., Lupu F., Ballard J., Metcalf J.P., James J.A., Farris D., Kurosawa S. (2013). The sepsis model: An emerging hypothesis for the lethality of inhalation anthrax. J. Cell Mol. Med..

[4] Doganay M., Metan G., Alp E. (2010). A review of cutaneous anthrax and its outcome. J. Infect. Public Health.

[5] Langer M., Malykhin A., Maeda K., Chakrabarty K., Williamson K.S., Feasley C.L., West C.M., Metcalf J.P., Coggeshall K.M. (2018). Bacillus anthracis peptidoglycan stimulates an inflammatory response in monocytes through the p38 mitogen-activated protein kinase pathway. PLoS ONE.

[6] Iyer J.K., Khurana T., Langer M., West C.M., Ballard J.D., Metcalf J.P., Merkel T.J., Coggeshall K.M. (2010). Inflammatory cytokine response to Bacillus anthracis peptidoglycan requires phagocytosis and lysosomal trafficking. Infect. Immun..

[7] Iyer J.K., Coggeshall K.M. (2011). Cutting edge: Primary innate immune cells respond efficiently to polymeric peptidoglycan, but not to peptidoglycan monomers. J. Immunol..

[8] Schleifer K.H., Kandler O. (1972). Peptidoglycan types of bacterial cell walls and their taxonomic implications. Bacteriol. Rev..

[9] Müller-Anstett M.A., Müller P., Albrecht T., Nega M., Wagener J., Gao Q., Kaesler S., Schaller M., Biedermann T., Götz F. (2010). Staphylococcal peptidoglycan co-localizes with Nod2 and TLR2 and activates innate immune response via both receptors in primary murine keratinocytes. PLoS ONE.

[10] Schwandner R., Dziarski R., Wesche H., Rothe M., Kirschning C.J. (1999). Peptidoglycan- and lipoteichoic acid-induced cell activation is mediated by toll-like receptor 2. J. Biol. Chem..

[11] Travassos L.H., Girardin S.E., Philpott D.J., Blanot D., Nahori M.A., Werts C., Boneca I.G. (2004). Toll-like receptor 2-dependent bacterial sensing does not occur via peptidoglycan recognition. EMBO Rep..

[12] Volz T., Nega M., Buschmann J., Kaesler S., Guenova E., Peschel A., Röcken M., Götz F., Biedermann T. (2010). Natural Staphylococcus aureus-derived peptidoglycan fragments activate NOD2 and act as potent costimulators of the innate immune system exclusively in the presence of TLR signals. FASEB J..

[13] de Jonge B.L., Chang Y.S., Gage D., Tomasz A. (1992). Peptidoglycan composition of a highly methicillin-resistant Staphylococcus aureus strain. The role of penicillin binding protein 2A. J. Biol. Chem..

[14] Langer M., Girton A.W., Popescu N.I., Burgett T., Metcalf J.P., Coggeshall K.M. (2018). Neither Lys- and DAP-type peptidoglycans stimulate mouse or human innate immune cells via Toll-like receptor 2. PLoS ONE.

[15] Sun D., Raisley B., Langer M., Iyer J.K., Vedham V., Ballard J.L., James J.A., Metcalf J., Coggeshall K.M. (2012). Anti-peptidoglycan antibodies and Fcγ receptors are the key mediators of inflammation in Gram-positive sepsis. J. Immunol..

[16] Popescu N.I., Cochran J., Duggan E., Kluza J., Silasi R., Coggeshall K.M. (2022). Internalization of Polymeric Bacterial Peptidoglycan Occurs through Either Actin or Dynamin Dependent Pathways. Microorganisms.

[17] Girardin S.E., Travassos L.H., Hervé M., Blanot D., Boneca I.G., Philpott D.J., Sansonetti P.J., Mengin-Lecreulx D. (2003). Peptidoglycan molecular requirements allowing detection by Nod1 and Nod2. J. Biol. Chem..

[18] Wolf A.J., Reyes C.N., Liang W., Becker C., Shimada K., Wheeler M.L., Cho H.C., Popescu N.I., Coggeshall K.M., Arditi M. (2016). Hexokinase Is an Innate Immune Receptor for the Detection of Bacterial Peptidoglycan. Cell.

[19] Irazoki O., Hernandez S.B., Cava F. (2019). Peptidoglycan Muropeptides: Release, Perception, and Functions as Signaling Molecules. Front. Microbiol..

[20] Geginat J., Nizzoli G., Paroni M., Maglie S., Larghi P., Pascolo S., Abrignani S. (2015). Immunity to Pathogens Taught by Specialized Human Dendritic Cell Subsets. Front. Immunol..

[21] van Beelen A.J., Zelinkova Z., Taanman-Kueter E.W., Muller F.J., Hommes D.W., Zaat S.A., Kapsenberg M.L., de Jong E.C. (2007). Stimulation of the intracellular bacterial sensor NOD2 programs dendritic cells to promote interleukin-17 production in human memory T cells. Immunity.

[22] Balraadjsing P.P., Lund L.D., Souwer Y., Zaat S.A.J., Frøkiær H., de Jong E.C. (2019). The Nature of Antibacterial Adaptive Immune Responses against Staphylococcus aureus Is Dependent on the Growth Phase and Extracellular Peptidoglycan. Infect. Immun..

[23] Gramlich R., Aliahmadi E., Peiser M. (2019). In Vitro Induction of T Helper 17 Cells by Synergistic Activation of Human Monocyte-Derived Langerhans Cell-Like Cells with Bacterial Agonists. Int. J. Mol. Sci..

[24] Zipperle G.F., Ezzell J.W., Doyle R.J. (1984). Glucosamine substitution and muramidase susceptibility in Bacillus anthracis. Can. J. Microbiol..

[25] Popescu N.I., Girton A., Burgett T., Lovelady K., Coggeshall K.M. (2019). Monocyte procoagulant responses to anthrax peptidoglycan are reinforced by proinflammatory cytokine signaling. Blood Adv..

[26] Munroe M.E., Vista E.S., Guthridge J.M., Thompson L.F., Merrill J.T., James J.A. (2014). Proinflammatory adaptive cytokine and shed tumor necrosis factor receptor levels are elevated preceding systemic lupus erythematosus disease flare. Arthritis Rheumatol..

[27] Sharif S., Singh M., Kim S.J., Schaefer J. (2009). Staphylococcus aureus peptidoglycan tertiary structure from carbon-13 spin diffusion. J. Am. Chem. Soc..

[28] Dixon T.C., Meselson M., Guillemin J., Hanna P.C. (1999). Anthrax. N. Engl. J. Med..

[29] Hahn A.C., Lyons C.R., Lipscomb M.F. (2008). Effect of Bacillus anthracis virulence factors on human dendritic cell activation. Hum. Immunol..

[30] Moayeri M., Leppla S.H., Vrentas C., Pomerantsev A.P., Liu S. (2015). Anthrax Pathogenesis. Annu. Rev. Microbiol..

[31] Stearns-Kurosawa D.J., Lupu F., Taylor F.B.J., Kinasewitz G., Kurosawa S. (2006). Sepsis and pathophysiology of anthrax in a nonhuman primate model. Am. J. Pathol..

[32] Popescu N.I., Silasi R., Keshari R.S., Girton A., Burgett T., Zeerleder S.S., Gailani D., Gruber A., Lupu F., Coggeshall K.M. (2018). Peptidoglycan induces disseminated intravascular coagulation in baboons through activation of both coagulation pathways. Blood.

[33] Sun D., Popescu N.I., Raisley B., Keshari R.S., Dale G.L., Lupu F., Coggeshall K.M. (2013). Bacillus anthracis peptidoglycan activates human platelets through FcγRII and complement. Blood.

[34] Gerosa F., Baldani-Guerra B., Lyakh L.A., Batoni G., Esin S., Winkler-Pickett R.T., Consolaro M.R., De Marchi M., Giachino D., Robbiano A. (2008). Differential regulation of interleukin 12 and interleukin 23 production in human dendritic cells. J. Exp. Med..

[35] Tada H., Aiba S., Shibata K., Ohteki T., Takada H. (2005). Synergistic effect of Nod1 and Nod2 agonists with toll-like receptor agonists on human dendritic cells to generate interleukin-12 and T helper type 1 cells. Infect. Immun..

[36] Kim H., Zhao Q., Zheng H., Li X., Zhang T., Ma X. (2015). A novel crosstalk between TLR4- and NOD2-mediated signaling in the regulation of intestinal inflammation. Sci. Rep..

[37] Khan N., Vidyarthi A., Pahari S., Negi S., Aqdas M., Nadeem S., Agnihotri T., Agrewala J.N. (2016). Signaling through NOD-2 and TLR-4 Bolsters the T cell Priming Capability of Dendritic cells by Inducing Autophagy. Sci. Rep..

[38] Harris K.M., Ramachandran G., Basu S., Rollins S., Mann D., Cross A.S. (2014). The IL-23/Th17 axis is involved in the adaptive immune response to Bacillus anthracis in humans. Eur. J. Immunol..

[39] Shieh W.J., Guarner J., Paddock C., Greer P., Tatti K., Fischer M., Layton M., Philips M., Bresnitz E., Quinn C.P. (2003). The critical role of pathology in the investigation of bioterrorism-related cutaneous anthrax. Am. J. Pathol..

[40] Grinberg L.M., Abramova F.A., Yampolskaya O.V., Walker D.H., Smith J.H. (2001). Quantitative pathology of inhalational anthrax I: Quantitative microscopic findings. Mod. Pathol..

[41] Ingram R.J., Ascough S., Reynolds C.J., Metan G., Doganay M., Baillie L., Williamson D.E., Robinson J.H., Maillere B., Boyton R.J. (2015). Natural cutaneous anthrax infection, but not vaccination, induces a CD4(+) T cell response involving diverse cytokines. Cell Biosci..

[42] Hedl M., Abraham C. (2012). IRF5 risk polymorphisms contribute to interindividual variance in pattern recognition receptor-mediated cytokine secretion in human monocyte-derived cells. J. Immunol..

[43] Lee M.N., Ye C., Villani A.C., Raj T., Li W., Eisenhaure T.M., Imboywa S.H., Chipendo P.I., Ran F.A., Slowikowski K. (2014). Common genetic variants modulate pathogen-sensing responses in human dendritic cells. Science.

[44] Fairfax B.P., Humburg P., Makino S., Naranbhai V., Wong D., Lau E., Jostins L., Plant K., Andrews R., McGee C. (2014). Innate immune activity conditions the effect of regulatory variants upon monocyte gene expression. Science.

